# Full‐zirconia single‐tooth molar implant‐supported restorations with angulated screw channel abutments: A 1‐year prospective case series study

**DOI:** 10.1111/cid.12872

**Published:** 2019-12-03

**Authors:** Christiaan W. P. Pol, Gerry M. Raghoebar, Zakelina Maragkou, Marco S. Cune, Henny J. A. Meijer

**Affiliations:** ^1^ Department of Implant Dentistry University of Groningen, University Medical Center Groningen Groningen The Netherlands; ^2^ Department of Oral and Maxillofacial Surgery University of Groningen, University Medical Center Groningen Groningen The Netherlands; ^3^ Department of Restorative Dentistry and Biomaterials University of Groningen, University Medical Center Groningen Groningen The Netherlands; ^4^ Department of Oral and Maxillofacial Surgery, Prosthodontics and Special Dental Care St. Antonius Hospital Nieuwegein Nieuwegein The Netherlands; ^5^ Department of Oral and Maxillofacial Surgery, Prosthodontics and Special Dental Care University of Utrecht, University Medical Center Utrecht Utrecht The Netherlands; ^6^ Department of Oral and Maxillofacial Surgery and Department of Implant Dentistry University of Groningen, University Medical Center Groningen Groningen The Netherlands

**Keywords:** angulated screw channel, dental implants, posterior, restoration, zirconia

## Abstract

**Background:**

Implant‐supported restorations in the posterior region are subjected to various complications that could be prevented by changing either the design or the material.

**Purpose:**

The aim of this prospective case series study was to evaluate full‐zirconia implant‐supported restorations with angulated screw channel abutments in the molar region of the maxilla and mandible and their effect on hard and soft peri‐implant tissues, during a 1‐year follow‐up period.

**Materials and Methods:**

Thirty consecutive patients with a single missing molar, sufficient bone height, and implant site free of infection were included. Each patient was to receive a parallel‐walled implant with conical connection according to a two‐staged surgical protocol. After 3 months, a full‐contour screw‐retained zirconia restoration with angulated screw channel abutment was provided. Clinical and radiographic examinations were performed 1 and 12 months after placement of the restoration. Patients' satisfaction was scored prior to treatment and after 12 months with the restoration in function. Primary outcome measure was success of the restoration.

**Results:**

All patients could be evaluated after 12 months. Success of the restorations was 100%. From loading to the 12‐month follow‐up, the mean marginal bone loss was 0.16 mm (SD: 0.26). Mean scores for plaque, calculus, peri‐implant mucosa, bleeding, and pocket probing depth were low, depicting healthy peri‐implant conditions. Patients' satisfaction was high and had improved after treatment.

**Conclusion:**

Full‐contour zirconia implant‐supported restorations with angulated screw channel abutments in the molar region have an excellent clinical performance after 1 year of function.

## 1 INTRODUCTION

Dental implant survival in cases of replacement of single missing teeth in the posterior region of the maxilla and mandible is high.^1^ However, implant‐supported restorations are prone to a number of complications, such as loosening of abutment screws and cement retention, peri‐implant mucosa infection due to cement remnants, and fracture of veneering ceramics.^2^, ^3^, ^4^, ^5^, ^6^, ^7^

Screw retention of implant restorations eliminates the risk of cement remnants and subsequent soft tissue complications.^8^ Loosening of screws could be minimized by an internal conical connection between implant and abutment.^9^ The possibilities for screw retention in the posterior region, however, can be hindered by off‐axis inclination or position of the implant when the location of the screw access opening of the restoration would be interfering with occlusion, articulation, or marginal thickness of the restoration. This could be of functional or aesthetic concern or could weaken the restoration; therefore, as an alternative to the traditional straight screw channel, the angulated screw channel has been developed.^10^ A recent prospective clinical study evaluating the retention of single restorations with angulated screw channel abutments has reported favorable results without major complications.^11^

Zirconia‐based implant‐supported single restorations are rated as highly successful with a cumulative 5‐year survival rate of 97.1%. However, the most common complication reported is the fracture of the veneering material, especially in the posterior region.^7^ The excellent mechanical properties of monolithic zirconia could help overcome this technical complication.^12^, ^13^ Nonetheless, there is only scarce clinical evidence in support of full‐contour zirconia implant‐supported single restorations,^7^, ^14^ while clinical evaluation of full‐contour zirconia restorations with angulated screw channel abutments has yet to be published. Also, the material properties of the restoration itself may have an impact on bone surrounding the implant. With finite element analysis, it has been calculated that an occlusal material with a high modulus of elasticity, such as zirconia, barely dampens occlusal impact forces, thereby increasing its effect on the bone‐implant interface.^15^, ^16^ Regarding soft tissues, it has been affirmed in systematic reviews that zirconia abutments have an excellent soft tissue response.^17^, ^18^ However, the impact of full‐zirconia restorations on soft tissue response has been addressed only in a limited number of studies.

Therefore, the aim of this prospective case series study was to evaluate full‐zirconia implant‐supported restorations with angulated screw channel abutments in the posterior region of maxilla and mandible, their effect on hard and soft peri‐implant tissues, and patients' satisfaction during a 1‐year follow‐up period.

## 2 MATERIALS AND METHODS

### 2.1 Patient enrolment

All patients referred to the Department of Oral and Maxillofacial Surgery (University of Groningen, University Medical Hospital, The Netherlands) from January 2016 till December 2016 for single‐tooth implant therapy in the maxillary and mandibular posterior region were considered for inclusion. The following inclusion criteria were applied:

- One missing tooth, being a first or second molar in the maxilla or mandible, with a minimum of 3 months of healing post‐extraction;
- Sufficient bone volume to insert a dental implant with a length of at least 7 mm;
- Implant site free from infection;
- Adequate oral hygiene (as expressed by modified plaque‐index^19^ and modified sulcus bleeding‐index^19^)
- Sufficient mesio‐distal, bucco‐lingual, and interocclusal space for placement of an anatomic restoration;
- Patient is capable of understanding and giving informed consent.

Patients were excluded from the experimental protocol when at least one of the following exclusion criteria was met:

- Medical and general contraindications for the surgical procedures;
- Presence of active and uncontrolled periodontal disease;
- Probable bruxism, based on self‐report and clinical examination, based on the consensus definition of Lobbezoo et al^20^;
- .Smoking: patient is declaring to be a smoker (and intends to continue) or has been smoking during the past 3 months;
- History of local radiotherapy to the head and neck region.

Patients fulfilling all the inclusion and none of the exclusion criteria were informed verbally and in writing about the study and signed the informed consent form.

The Medical Ethical Committee of the University Medical Center Groningen considered this case series study not to be subject to the Medical Research Involving Human Subjects Act (Number M15.184100).

## 3 SURGICAL AND PROSTHETIC PROCEDURES

The surgical and prosthetic treatments were performed at the Department of Oral and Maxillofacial Surgery, University Hospital Groningen. One oral surgeon, experienced in implant dentistry, executed the surgical treatments and two experienced prosthodontists performed restorative procedures. All laboratory procedures have been carried out in a single dental laboratory.

### 3.1 Surgical procedure

At the time of intervention, there was a healed site with a missing tooth for more than 3 months. There was enough bone to reach primary implant stability. One hour preoperatively antibiotic prophylaxis (2 g amoxicillin or, if allergic to penicillin, 600 mg clindamycin) was given and a 0.2% chlorhexidine mouthwash (two times daily for 10 days) was prescribed for oral disinfection. The surgical procedure was performed under local anesthesia. A parallel‐walled implant with a TiUnite surface and conical connection (NobelParallel CC, Nobel Biocare AB, Goteborg, Sweden) was placed, according to the manufacturers' protocol. Implant diameter was 4.3 mm and length varied from 8.5 to 13 mm, dependent on available bone height at the implant site. A cover screw (Nobel Biocare AB) was placed, and the wound was closed. One week after implant placement, a follow‐up visit was scheduled for suture removal and review of the healing process. After 3 months, the implant was uncovered and a healing abutment (Healing Abutment CC RP, Nobel Biocare AB) was installed.

### 3.2 Restorative procedure

**Figure 1:**
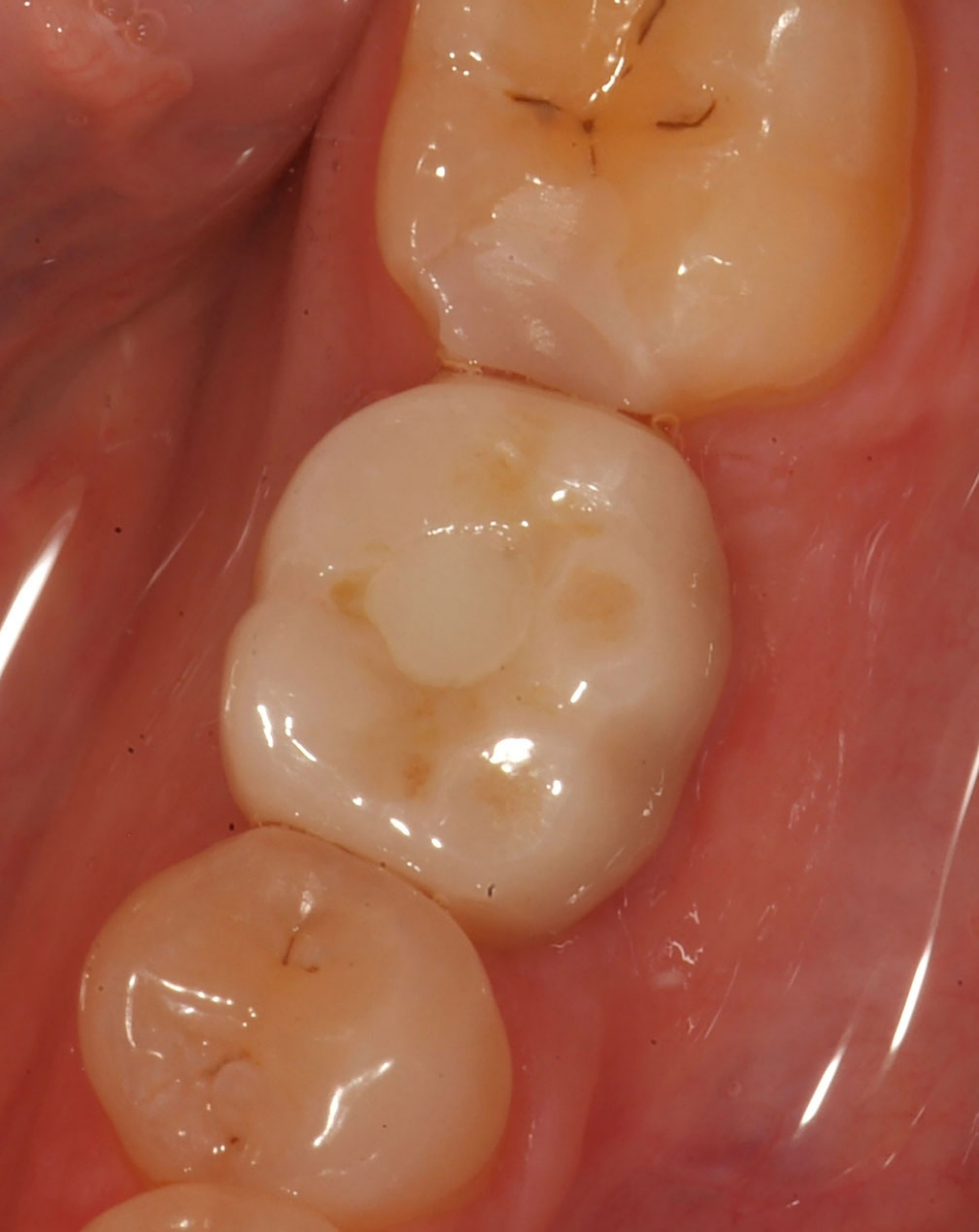
Full‐contour zirconia screw‐retained restoration at position 36

**Figure 2:**
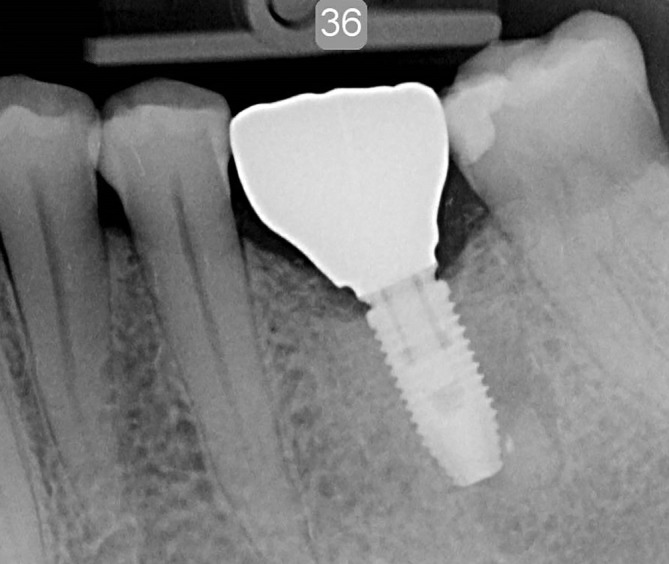
Intraoral radiograph of a parallel‐walled implant with a full‐contour zirconia restoration and angulated screw channel abutment

An impression at implant level (Impression coping open tray CC RP, Nobel Biocare AB) was taken 2 weeks after second stage surgery for fabrication of a single crown. The color of the future crown was determined using the scale of eight possible shades for zirconia delivered by the company (Nobel Biocare AB). In the dental laboratory, a dental cast with implant analogue was made and digitized with a dental laboratory scanner (Ceramill Map 400, Amann Girrbach AG, Koblach, Austria). A full‐contour crown was designed with dedicated design software (Ceramill Mind, Amann Girrbach) and subsequently milled as complete contour wax pattern (Ceramill Motion, Amann Girrbach). The custom wax pattern was scanned (NobelProcera 2G Scanner, Nobel Biocare AB) duplicating the design for a full‐zirconia crown (yttria‐stabilized zirconium oxide) allowing an angulated screw channel (NobelProcera FCZ Implant Crown and with ASC feature, Nobel Biocare AB) with design software (NobelProcera, NobelBiocare AB). The crown was manufactured in the determined color at a centralized milling facility (NobelProcera Service Center, Mahwah, New Jersey) and additionally stained and glazed at the dental laboratory for achieving the final color (Ceram Essence and Ceram Glaze Paste, Ivoclar Vivadent, Schaan, Liechtenstein). The abutment and crown were assembled and screw retained onto the implant with a torque of 35 Ncm. The screw access hole was sealed with a cotton pellet and light‐curing composite material (Figures 1 and 2).

Immediately after placement of the restoration, thorough oral hygiene instructions were given to all patients.

## 4 OUTCOME MEASURES

Primary outcome measure was success of the restoration, ascertained following modified United States Public Health Service (USPHS) criteria, being a composition of the outcomes: fracture of framework, loosening of restoration, wear facets, catching of probe at margin, anatomical shape, mismatch in color shade, cementation gap on radiograph, and patient satisfaction.^21^, ^22^

Clinical and radiographic evaluation was performed 1 month and 1 year after placement of the restoration. The following assessments were made:

- Implant survival. The survival rate of the implant was assessed 1 year after placement of the definitive restoration. Implant failure was defined when removal of the implant was deemed necessary because of implant mobility as a consequence of insufficient or lost osseointegration;
- Marginal bone level as measured on standardized intraoral radiographs;
- Assessment of plaque accumulation with the modified Plaque Index^19^;
- Assessment of bleeding tendency with the modified Sulcus Index^19^;
- Assessment of peri‐implant inflammation with the Gingival Index^23^;
- Presence of calculus
- Probing pocket depth: measured to the nearest millimeter using a manual periodontal probe (Williams‐Sulcus color‐coded probe, Hu‐Friedy, Chicago, Illinois). Probing of the implant was performed at four sites (mesial, distal, buccal, and lingual/palatinal);
- Restoration survival;
- Complications related to the restoration;
- Patients' satisfaction. Patients were asked to complete a questionnaire prior to implant placement and 1 year after placement of the restoration.

With regard to the radiographic evaluation, radiographs were taken 1 and 12 months after restoration placement using a parallel technique, with an X‐ray holder for periapical radiographs. The radiographs were analyzed using dedicated computer software to perform linear measurements on the digital radiographs. The calibration was carried out in the vertical plane for each radiograph by using the known length of the implant and distance between several threads. This calibration ensured a correct measurement.^24^ Crestal bone changes were determined by measuring, both mesially and distally, the distance from the reference line to the level of the margin of the crestal bone. The reference line on the radiograph was constructed by intersecting the upper most top of the implant neck on the mesial and distal edge. Bone levels above the reference line were considered to be zero to eliminate the maturation of the bone after subcrestal placement. Bone loss was presented as the worst value of either distal or mesial change between 1 and 12 months after restoration placement for each implant.

The patients' satisfaction was recorded by means of a questionnaire asked to be filled out by the patients before surgery and 1 year after restoration placement. The nonvalidated questionnaire, previously published by Telleman 2013,^25^ comprised of questions or statements to be answered on a 5‐point rating scale ranging from “very dissatisfied”/“not in agreement” (score 1) to “very satisfied”/“in agreement” (score 5). The topics addressed were related to aesthetics and appearance, function (chewing), sense (“feeling like natural teeth”), speech, and self‐esteem. Furthermore, patients were asked to mark their overall satisfaction concerning their dental situation at time of enrolment and at the 1‐year evaluation on a 10‐point rating scale from 0 to 10, in which 10 is the highest satisfaction score.

## 5 STATISTICAL ANALYSIS

One observer was responsible for the collection and analysis of all the data. The worst score of the clinical and radiographic parameters evaluated per implant was used in the data analysis. Data were presented as frequencies. Differences in patients' satisfaction between pretreatment and 1‐year follow‐up were tested with the Wilcoxon signed rank test. Analysis was done with PASW Statistics 23.0 (SPSS Inc., an IBM Company, IBM Corporation, Chicago, Illinois). In all tests, the significance level *α* was set to 5%.

## 6 RESULTS

All consecutive patients eligible to join the study on the basis of the inclusion and exclusion criteria agreed to participate in this study. A total of 30 patients (10 males and 20 females, mean age 53 years, range 27‐83 years) who were to receive 30 implants were included. Patient characteristics are depicted in Table 1. All patients completed the 1‐year evaluation period. Implant and restoration survival were 100% at the 12‐month evaluation.

**Table 1:** Baseline characteristics of study group

Number of participants (patients/implants)	30/30
Mean age in years (SD, minimum‐maximum)	53 (13.3, 27‐83)
Gender (number male/female)	10/20
Implant position (maxilla/mandible)	12/18
Implant position (in between teeth/no tooth distally)	24/6

The mean scores of the indices for plaque, calculus, gingiva, and bleeding were very low, indicating favorable results (Table 2). The mean probing depth was 1.7 mm (SD 0.8 mm) at the 1‐year follow‐up. The mean loss of marginal bone between 1 month after restoration placement (*T*_1_) and 1‐year postloading (*T*_12_) was 0.16 mm (SD 0.26 mm) (Table 3).

**Table 2:** Frequencies and percentages of plaque‐index scores (possible score 0‐3), calculus‐index scores (possible score 0‐1), gingival‐index scores (possible score 0‐3), bleeding‐index scores (possible score 0‐3), and mean value, SD of probing depth (in mm) 1 month after placement of restoration (*T*_1_) and after 1 year (*T*_12_)

	*T*_1_	*T*_12_
Plaque‐index	Score 0: 30 (100%)	Score 0: 30 (100%)
Calculus‐index	Score 0: 30 (100%)	Score 0: 30 (100%)
Gingival‐index	Score 0: 30 (100%)	Score 0: 30 (100%)
Bleeding‐index	Score 0: 24 (80%) Score 1: 6 (20%)	Score 0: 27 (90%) Score 1: 3 (10%)
Probing depth in mm (SD)	2.0 (0.6)	1.7 (0.8)

**Table 3:** Mean value and SD and frequency distribution (percentages) of marginal bone change between 1 month after restoration placement (*T*_1_) and 1 year in function (*T*_12_), based on lowest value per implant

Bone change (mm)	n = 30
Mean (SD)	−0.16 mm (0.26)
> −1.5 till −1.0	1 (3.3%)
> −1.0 till −0.5	1 (3.3%)
> −0.5 till 0.0	28 (93.4%)

Patient's satisfaction had significantly improved at the 1‐year evaluation (*P* < .001). Mean presurgical overall score was 6.1 ± 0.7 at a scale of 1 to 10, and after treatment, the mean score was 9.0 ± 0.8. Feelings of shame because of visibility of being partially edentulous decreased (*P* < .001). Patients were significantly more satisfied about the ability to chew after restoration (*P* < .001) (Table 4). The quality of the restoration was assessed according to modified USPHS criteria. All applicable parameters were without concerns and favorable, resulting in a 100% restoration success (Table 5).

**Table 4:** Patient's satisfaction before treatment (*T*_pre_), after 12 months (*T*_12_), and significant differences between time periods

	*T*_pre_ % in agreement	*T*_12_ % in agreement	*P* value
	(n = 30)	(n = 30)	
Feelings			
Presence of shame	26.7	0.0	*P* < .001
Self‐confidence decreased	0.0	0.0	*P* = 1.000
Visible being partial edentulous	43.3	0.0	*P* < .001
Function			
Evade eating with the edentulous zone/implant	63.3	3.3	*P* < .001
The ability to chew is decreased	43.3	0.0	*P* < .001
Implant does influence the speech	—	0.0	
Implant does influence the taste	—	0.0	
Aesthetics			
not satisfied with the color of the crown	—	0.0	
not satisfied with the form of the crown	—	0.0	
not satisfied with the color of the mucosa around the crown	—	0.0	
not satisfied with the form of the mucosa around the crown	—	0.0	
Overall satisfaction (0‐10)	6.1 ± 0.7	9.0 ± 0.8	*P* < .001

**Table 5:** USPHS criteria for evaluation of the restoration at 1‐year follow‐up

USPHS criteria	Alpha (A)	Bravo (B)	Charlie (C)	Delta (D)
Framework fracture	No fracture of framework, 30 (100%)	—	—	Fracture of framework, 0 (0%)
Veneering fracture	No fracture, not applicable	Chipping but polishing possible, not applicable	Chipping down to framework, not applicable	New reconstruction is mandatory, not applicable
Loosening of the restoration (cement and/or screw)	No loosening, 30 (100%)	—	Repositioning possible, 0 (0%)	Repositioning not possible—new reconstruction is needed, 0 (0%)
Screw access hole restoration	No loss of restoration, 30 (100%)	—	Restoration lost (repairable), 0 (0%)	—
Occlusal wear	No wear facets on restoration and opposing teeth, 30 (100%)	Small wear facets (diameter < 2 mm) on restoration and/or opposing teeth, 0 (0%)	Wear facets (diameter > 2 mm) on restoration and/or opposing teeth, 0 (0%)	New reconstruction is needed, 0 (0%)
Marginal adaptation	Probe does not catch, 30 (100%)	Probe catches slightly, but no gap detectable, 0 (0%)	Gap with dentin or cement exposure, 0 (0%)	New reconstruction is needed, 0 (0%)
Anatomical form	Ideal anatomical shape, good proximal contacts, 30 (100%)	Slightly over‐ or undercontoured, weak proximal contacts, 0 (0%)	Highly over‐ or undercontoured, open proximal contacts, 0 (0%)	New reconstruction is needed, 0 (0%)
Restoration color	No mismatch in color shade between restoration and adjacent teeth, 0 (0%)	Slight mismatch between color shade of restoration and adjacent teeth, 30 (100%)	Mismatch between restoration and adjacent teeth outside normal range of color shade, 0 (0%)	Shade in gross disharmony with adjacent teeth—new reconstruction is needed, 0 (0%)
Radiographs	No visible cementation gap on X‐ray, not applicable	Minor gap visible, not applicable	Major gap visible—new reconstruction not needed, not applicable	Major gap visible—new reconstruction is needed, not applicable
Patient satisfaction	Very satisfied, 30 (100%)	Moderately satisfied, 0 (0%)	Not satisfied—new reconstruction not needed, 0 (0%)	Not satisfied—new reconstruction needed, 0 (0%)
Lowest value per restoration	**Success and survival**, 0 (0%)	**Success and survival**, 30 (100%)	**Survival, no success**, 0 (0%)	**Failure**, 0 (0%)

## 7 DISCUSSION

Full‐contour zirconia implant‐supported restorations with angulated screw channel abutments in the posterior region exhibited an outstanding performance with a 1‐year restoration success of 100% according to the USPHS criteria, paired with a 100% survival of the implants. No complications were noticed, such as fracture of the framework, loosening of the restoration, or occlusal wear. The performance of parallel‐walled implants with conical connection in combination with restorations with an angulated screw channel abutment was recently reported by Friberg and Ahmadzai as well.^11^ They reported an implant survival rate reaching 98% and a restoration survival of 100% after 1 year in function. It must be noted that in the latter study, zirconia‐based porcelain veneered restorations were used, but the reported excellent survival rates are in line with the present study. At this moment, no studies with full‐zirconia restorations and angulated screw channel abutments in the posterior region have been published, which precludes a direct comparison with the literature.

Limited peri‐implant bone loss was found at the 1‐year evaluation, being somewhat less compared to the data from the aforementioned study by Friberg and Ahmadzai,^11^ being 0.16 vs 0.41 mm. Therefore, it could be hypothesized that this new implant design with the conical internal connection creates favorable conditions in the implant restoration interface that minimize posttreatment marginal bone loss. Ideally, the initial bone height should be assessed on radiographs taken immediately after loading. In this study, the first radiograph was taken 4 weeks after loading, during the first clinical examination.

Zirconia is presumed to be highly biocompatible with a potential for soft tissue attachment.^26^ With regard to the evaluation of peri‐implant soft tissues, the findings of the present study were consistent with a healthy status and confirming the high biocompatibility of the material. The limited probing depth (mean value of 1.7 mm) is possibly associated with the proposed soft tissue attachment potential. It has to be acknowledged that the probing depth around implant‐supported restorations is difficult to measure due to the convex contour, soft tissue health was, however, also affirmed by the low bleeding‐index and excellent gingiva‐index. An advantage of screw‐retained restorations, with either straight or angulated screw channel, is the absence of a microgap at the interface of crown and abutments and the absence of possible cement remnants in the area of the peri‐implant soft tissues. In addition, the high patient compliance to the posttreatment oral hygiene instructions prescribed could also have played an important role in the observed very healthy peri‐implant soft tissues.

In an attempt to incorporate the concept of patient engagement, this study sought to investigate patients' satisfaction in relation to the rehabilitated posterior region by assessing specific patient‐centered outcomes. This was done by having the patients fill out an established questionnaire prior to implant placement and 1 year after the placement of the restorations.^25^, ^27^ Similar to the high level of satisfaction reported in comparable studies with single tooth replacement in the posterior region and using the same questionnaires,^25^, ^27^ all assessed outcome measures had significantly improved after treatment. Therefore, in addition to the excellent clinical performance, high patient acceptance of the implant‐supported full‐zirconia single posterior crowns was affirmed. In these two studies, zirconia‐based porcelain veneered restorations were used as opposed to monolithic zirconia in the present study. With monolithic zirconia, the dental laboratory has limited possibility to individually design and finish the restoration compared to porcelain veneering. Apparently, the slight mismatch between the color shade of the restoration and the adjacent teeth, observed by the trained observer, did not affect the patients' satisfaction because none of them was dissatisfied with the color of the restoration. Also, the composite restoration in the occlusal surface to seal the screw channel did not impact the patients' opinion. This might be caused by the fact that patients are valuing the rehabilitation of an extraction gap in the posterior region over aesthetics of the restoration.

Single‐tooth implant prosthodontics using full‐zirconia restorations, an angulated screw channel feature, and a conical connection to replace a missing molar results in favorable treatment outcomes biologically, technically, and subjectively. However, the small sample size should be acknowledged, as the herein described clinical protocol was performed on a limited number of patients. In addition, the inherent lack of a control group associated with a case series study is another factor that needs to be taken into account. In most cases, the angle of the screw channel was small; the results might be different when using this abutment only in more extreme angled cases. The results from this study cannot be used to predict results of the same treatment strategy when used in the anterior region, where the forces might differ significantly and more challenging angulations might be involved. Furthermore, the 1‐year follow‐up period, even though indicative for early implant failures and short‐term restorative complications, is considered short as posttreatment evaluation period. Long‐term assessments of a larger study population are therefore warranted in order to verify over time the outstanding clinical performance observed. Further research should also address more challenging cases, such as abutments with more angled screw channels, and more challenging patients, such as bruxers or smokers, now that it has been established that the protocol works in less challenging circumstances. Lastly, the absence of occlusal wear of the antagonists noted in the present study was confirmed on the macroscopic level. Despite the high clinical relevance of this outcome measure, a long‐term in‐depth analysis of the induced wear on the antagonists on the microscopic level could provide clinically meaningful data.

## 8 CONCLUSIONS

Within the limitations of this study, it has been demonstrated that full‐contour implant‐supported zirconia restorations with angulated screw channel abutments in the molar region have an excellent clinical performance after 1 year of function.

## CONFLICT OF INTEREST

The authors have stated explicitly that there are no conflicts of interest in connection with this article.
